# Apnea Testing on Conventional Mechanical Ventilation During Brain Death Evaluation

**DOI:** 10.1007/s12028-024-01990-8

**Published:** 2024-04-25

**Authors:** Rameez Ali Merchant, Shahid Nafees Ahmad, Bradley Haddix, Craig Andrew Williamson, Teresa Lee Jacobs, Tarun Deep Singh, Andrew M. Nguyen, Venkatakrishna Rajajee

**Affiliations:** 1 Department of Neurosurgery, University of Michigan, 3552 Taubman Health Care Center, SPC 5338, 1500 E. Medical Center Drive, Ann Arbor, MI 48109-5338 USA; 2 Department of Neurology, University of Michigan, Ann Arbor, MI USA; 3 Southern Hills Hospital and Medical Center, Las Vegas, NV USA; 4 Department of Respiratory Care, University of Michigan, Ann Arbor, MI USA

**Keywords:** Brain death, Apnea, Mechanical ventilation

## Abstract

**Introduction:**

The use of continuous positive airway pressure has been shown to improve the tolerance of the apnea test, a critical component of brain death evaluation. The ability to deactivate the apnea backup setting has made apnea testing possible using several conventional mechanical ventilators. Our goal was to evaluate the safety and efficacy of apnea testing performed on mechanical ventilation, compared with the oxygen insufflation technique, for the determination of brain death.

**Methods:**

This was a retrospective study. In 2016, our institution approved a change in policy to permit apnea testing on conventional mechanical ventilation. We examined the records of consecutive adults who underwent apnea testing as part of the brain death evaluation process between 2016 and 2022. Using an apnea test technique was decided at the discretion of the attending physician. Outcomes were successful apnea test and the occurrence of patient instability during the test. This included oxygen desaturation (SpO2) < 90%, hypotension (mean arterial pressure < 65 mm Hg despite titration of vasopressor), cardiac arrhythmia, pneumothorax, and cardiac arrest.

**Results:**

Ninety-two adult patients underwent apnea testing during the study period: 58 (63%) with mechanical ventilation, 32 (35%) with oxygen insufflation, and 2 (2%) lacked documentation of technique. Apnea tests could not be completed successfully in 3 of 92 (3%) patients—two patients undergoing the oxygen insufflation technique (one patient with hypoxemia and one patient with hypotension) and one patient on mechanical ventilation (aborted for hemodynamic instability). Hypoxemia occurred in 4 of 32 (12.5%) patients with oxygen insufflation and in zero patients on mechanical ventilation (*p* = 0.01). Hypotension occurred during 3 of 58 (5%) tests with mechanical ventilation and 4 of 32 (12.5%) tests with oxygen insufflation (*p* = 0.24). In multivariate analysis, the use of oxygen insufflation was an independent predictor of patient instability during the apnea test (odds ratio 37.74, 95% confidence interval 2.74–520.14).

**Conclusions:**

Apnea testing on conventional mechanical ventilation is feasible and offers several potential advantages over other techniques.

## Introduction

The apnea test is a critical component of brain death evaluation, to establish the absence of spontaneous respiratory effort, despite an adequate carbon dioxide stimulus [1]. The apnea test has traditionally been performed with disconnection from mechanical ventilation and insufflation of oxygen through a cannula in the endotracheal tube (ETT) while observing for spontaneous respiratory effort [1]. Several important challenges have been identified with the conduct of apnea tests [2]. The removal of positive pressure with this technique may lead to atelectasis, oxygen desaturation, and hemodynamic instability, particularly in patients with severe hypoxemic respiratory failure or severe left ventricular systolic dysfunction [3–5]. Occlusion of the ETT by the oxygen cannula may lead to hyperinflation of the lungs and pneumothorax [5, 6]. The use of a continuous positive airway pressure (CPAP) valve with improvised equipment has been shown to improve the tolerance of apnea testing [7, 8]. While superior to oxygen insufflation, these improvised CPAP techniques have several limitations. Additional equipment is required, such as a T-piece and a CPAP valve. Patients with the acute respiratory distress syndrome (ARDS) often require higher levels (> 10 cm H_2_O) of positive end-expiratory pressure (PEEP) than improvised CPAP circuits can provide, and they may suffer derecruitment with the brief disconnection required to switch tubing. In contrast, performance of the apnea test with the mechanical ventilator that is already in use does not require special equipment or disconnection, and high PEEP levels can be maintained. Even minimal spontaneous inspiratory effort, which can be challenging to detect through visual observation of the chest wall alone, can be quickly and easily detected on the ventilator’s scalar waveforms. Although the presence of a required apnea backup setting that forces resumption of breath delivery was previously an obstacle to apnea testing using conventional ventilators, this backup setting may be turned off in several contemporary machines. A 2019 Polish study demonstrated feasibility of this technique in patients already declared brain dead [9]. Although ventilator-based apnea testing was recently endorsed by American Academy of Neurology and World Brain Death Project guidelines [1, 10], real-world clinical validation during brain death determination is required. The success rate in patients with ARDS with poor oxygenation status is uncertain, and concerns exist about autotriggering or artifactual respiratory effort.

Therefore, our objective was to study the rate of successful completion and occurrence of complications with the use of conventional mechanical ventilation, compared to the oxygen insufflation technique, for apnea tests performed during brain death evaluation.

## Methods

The University of Michigan Institutional Review Board determined that this retrospective observational study that included data acquired prospectively for quality assurance (QA) purposes (HUM00163494) was exempt from regulation. The records of consecutive adults (age ≥ 18 years) who underwent a complete brain death evaluation from July 2016 through September 2022 were reviewed. Patients were identified on the basis of an institutional brain death registry, which includes all patients who have undergone a complete brain death evaluation. Baseline characteristics, including primary diagnosis, ratio of partial pressure of arterial oxygen to fraction of inspired oxygen (P/F ratio) measured before the apnea test, presence of ARDS based on the Berlin criteria [11], ventilator settings before the apnea test, vasopressor requirement, coronavirus disease 2019 (COVID-19) status, use of venovenous (VV) or venoarterial (VA) extracorporeal membrane oxygenation (ECMO), and organ donor status, were extracted from institutional medical records. Variables related to the apnea test, such as technique (oxygen insufflation vs. conventional mechanical ventilation), duration of the test, and peak arterial partial pressure of carbon dioxide (pCO_2_) were reviewed.

### Apnea Test Technique

Brain death evaluation was performed in concordance with institutional policy. In 2016, following the acquisition of a fleet of Dräger Evita Infinity V500 ventilator machines, which provide an option to turn off the apnea backup, and clinical experience outside the brain death evaluation process, institutional policy was modified to allow performance of the apnea test on conventional mechanical ventilation. A postapproval neurocritical care QA project was initiated to monitor the use of this technique. Between July 2016 through December 2018, all apnea tests conducted on mechanical ventilation were supervised by neurointensivists who monitored ventilator scalars and reported any spontaneous or artifactual respiratory effort. Based on the results of this QA assessment, routine neurointensivist monitoring of apnea tests conducted on mechanical ventilation was discontinued in 2019, although the presence of a physician continued to be a requirement of the longstanding institutional protocol. The protocol also requires that details of any patient instability, artifactual respiration, or evidence of spontaneous respiration be included in brain death documentation within the medical record. Choice of apnea test technique was at the discretion of the attending physician. Preoxygenation was performed for ≥ 10 min before the apnea test. The target duration of the test was 10 min, at which point arterial blood gas analysis was performed. The test was terminated sooner in the presence of spontaneous respiratory effort or cardiopulmonary instability, defined as an oxygen saturation (SpO2) level < 90%, mean arterial pressure (MAP) < 65 mm Hg despite titration of vasopressor, systolic blood pressure < 90 mm Hg despite titration of vasopressor, or new cardiac arrhythmia. A decision to terminate the apnea test for severe patient instability was at the discretion of the physician supervising the test. When the apnea test was terminated for cardiopulmonary instability, arterial blood gas testing was performed before resumption of machine-initiated ventilation. Institutional criteria for an adequate respiratory stimulus were pCO_2_ ≥ 60 mm Hg with a ≥ 20-mm Hg rise from baseline. Apnea tests on mechanical ventilation were performed with Dräger Evita Infinity V500 machines in pressure support mode with the apnea backup turned off. The process to turn off the apnea backup in this and other commonly used conventional ventilators is presented in Table 1. Pressure support was turned to zero and variable PEEP, appropriate to the individual patient, was used. Apnea tests with oxygen insufflation were performed with disconnection from the ventilator, insertion of oxygen tubing into the ETT, and oxygen flow at 4–6 L/min. Patients who could not undergo or successfully complete apnea testing required a confirmatory test for the determination of brain death.

**Table 1:** Ability to disable the apnea backup setting: ventilator models

Ventilators	Apnea backup can be turned off	How to turn Apnea backup off (In a mode with apnea backup)
Drager Evita Infinity V500	Yes	Select “Ventilation Settings,” select “Additional Settings,” select “Apnea Ventilation,” select “off,” confirm with control knob
Drager Evita V800	Yes	Select the button with three dots for additional settings, select “Apnea Ventilation,” select “off,” confirm with control knob
Getinge Servo-i	Yes, if enabled in Biomed menu	Select “Backup Ventilation” in the set ventilation mode window. A confirmation dialog “Do you really want to deactivate backup ventilation?” is displayed. Confirm by pressing “Yes.” Press “Accept” in the set ventilation mode window
Getinge Servo-u	Yes, if enabled in Biomed menu	Select “Deactivate Backup Ventilation” in the mode settings window. A confirmation dialog “Do you really want to deactivate backup ventilation?” is displayed. Confirm by pressing “Yes.” Tap “Accept” in mode settings window
GE HealthCare Carescape R860	Yes	Select “Current Mode,” select “Mode Settings,” uncheck the “Backup Mode” box
Hamilton-C6, C3, C1	Yes	Select “Controls,” select “Apnea,” uncheck “Backup”
Hamilton-G5	Yes	Select “Controls,” uncheck “Backup”
Medtronic Puritan Bennett 840	No	
Medtronic Puritan Bennett 980	No	
Vyaire Avea	No	
Vyaire Bellavista 1000	Yes	Select the backup ventilation screen dot, select “Backup Mode,” select “Backup off.”

Disclaimer: Information is subject to change with future ventilator software updates

### Outcomes

The primary outcome of interest was successful completion of the apnea test. The secondary outcome of interest was the occurrence of any complications attributable to apnea testing, including but not limited to institutional criteria for discontinuation: oxygen desaturation (SpO2 < 90%), hypotension (MAP < 65 mm Hg) despite titration of vasopressors, pneumothorax, new cardiac arrhythmia, and cardiac arrest. We also examined the occurrence of autotriggering events (with flow trigger at 2 L/min) and any description of artifactual waveforms that resembled spontaneous respiratory effort.

### Statistical Analysis

Descriptive analysis was performed with proportions and percentages for categorical variables and medians with interquartile ranges (IQRs) for continuous variables. The statistical significance of associations between variables and outcomes of interest was assessed with the *χ*^2^ test or Fischer exact test for categorical variables and the Mann–Whitney *U*-test for continuous variables. Multivariate analysis was performed with patient instability of any cause as the binary response variable and the following predictor variables: age, cause of brain death, *P*/*F* ratio, vasopressor requirement, use of ECMO, and apnea test technique. Odds ratios (OR) with 95% confidence intervals (CIs) were calculated for independent predictors of the outcome. The threshold for statistical significance was *p* < 0.05.

## Results

A total of 92 adult patients underwent apnea testing as part of the brain death evaluation process during the study period. Of these, 58 (63%) patients were tested with conventional mechanical ventilation, 32 (35%) patients were tested with oxygen insufflation, and data from 2 (2%) patients lacked documentation of the apnea test technique used. Thirty-six adult patients underwent apnea testing in the 2016–2018 period of QA and direct neurointensivist monitoring. Of these, 17 (47%) patients were tested with conventional mechanical ventilation and 19 (53%) patients were tested with oxygen insufflation. In the 2019–2022 period, 56 patients underwent apnea testing. Of these, 41 (73%) patients were tested with conventional mechanical ventilation, 13 (23%) patients were tested with oxygen insufflation, and 2 (2%) patients lacked documentation of the apnea test technique. Baseline characteristics of all patients are in Table 2. Berlin criteria for ARDS were present in 22 of 92 (24%) patients; of these, 18 of 22 (82%) patients underwent the apnea test on mechanical ventilation, 3 of 22 (14%) underwent oxygen insufflation, and 1 of 22 (5%) lacked documentation of the apnea test technique used (*p* = 0.02). Overall, 36 of 92 (39%) patients had acute hypoxemic respiratory failure with a *P*/*F* ratio < 300 mm Hg, and 19 of 92 (21%) patients had a *P*/*F* ratio < 200 mm Hg. The median PEEP before the apnea test was 5 mm Hg (IQR 5–8 mm Hg, range 5–22 mm Hg). Vasopressor support was required in 49 of 58 (85%) patients tested with mechanical ventilation and in 27 of 32 (84%) with oxygen insufflation (*p* = 0.99). Four patients were on ECMO at the time of attempted apnea testing—three on VA-ECMO and one on VV-ECMO. Apnea tests were performed on the ventilator for all four patients. Of these, testing was successfully completed in all three patients on VA-ECMO. Four patients who underwent apnea testing during the pandemic had COVID-19 ARDS. Of these, three had suffered cardiac arrest and hypoxemic-ischemic injury as the cause of brain death, whereas the fourth had suffered a large intracerebral hemorrhage. All patients with COVID-19 were tested on mechanical ventilation.

**Table 2:** Baseline characteristics

Variable	All patients (*N* = 90)	Apnea test with oxygen insufflation (*n* = 32)	Apnea test with conventional mechanical ventilation (*n* = 58)	*p* value
Age in years, median (IQR)	39 (25–53)	38 (25–50)	48 (35–59)	0.007
Female	34 (38%)	10 (31%)	24 (41%)	0.34
Cause of brain death				0.59
Cardiac arrest	30 (33%)	13 (41%)	17 (29%)	
SAH	16 (18%)	2 (6%)	14 (24%)	
TBI	14 (16%)	6 (19%)	8 (14%)	
Ischemic stroke	14 (16%)	5 (16%)	9 (16%)	
ICH	8 (9%)	3 (9%)	5 (9%)	
Other	8 (9%)	3 (9%)	5 (9%)	
FiO_2_, median (IQR)	0.4 (0.3–0.55)	0.4 (0.33–0.55)	0.4 (0.3–0.55)	0.16
PEEP in cmH_2_O, median (IQR)	5 (5–8)	5 (5–8)	8 (5–10)	0.55
*P*/*F* ratio in mmHg, median (IQR)	382 (223–820)	464 (279–881)	343 (210–780)	0.14
ARDS present	21 (23%)	3 (9%)	18 (31%)	0.02
COVID-19 ARDS	4 (4%)	0 (0%)	4 (7%)	
Vasopressor infusion				
Any	76 (84%)	27 (84%)	49 (85%)	0.99
Norepinephrine	58 (64%)	22 (69%)	36 (62%)	
Vasopressin	50 (56%)	16 (50%)	34 (59%)	
Dopamine	2 (2%)	0 (0%)	2 (3%)	
ECMO	4 (4%)	0 (0%)	4 (7%)	0.29
Venoarterial	3 (3%)		3 (5%)	
Venovenous	1 (1%)		1 (2%)	
Organ donor	60 (67%)	21 (66%)	39 (67%)	1.00

*ARDS* acute respiratory distress syndrome, *ECMO* extracorporeal membrane oxygenation, *FiO2* fraction of inspired oxygen, *ICH* intracerebral hemorrhage, *IQR* interquartile range, *P/F* ratio of partial pressure of oxygen to fraction of inspired oxygen, *PEEP* positive end expiratory pressure, *SAH* subarachnoid hemorrhage, *TBI* traumatic brain injury

Overall, 3 of 92 (3%) attempted apnea tests could not be completed successfully because of patient instability—two with the oxygen insufflation technique and one on mechanical ventilation. Of the two unsuccessful apnea tests performed with oxygen insufflation, one each required premature termination as a result of oxygen desaturation to SpO2 < 90% (patient with high-grade subarachnoid hemorrhage and ARDS) and hypotension to MAP < 65 mm Hg despite titration of vasopressor (patient with polytrauma and severe traumatic brain injury on vasopressors). The single patient with an unsuccessful apnea test on mechanical ventilation had suffered hypoxemic cardiac arrest from COVID-19 ARDS and was on VV-ECMO. Termination of the test was for severe hemodynamic instability. In all three patients, brain death was declared following confirmatory testing with radionuclide cerebral perfusion scans. The median duration of the apnea test was 10 min (IQR 10–12 min) on the ventilator versus 10 min (IQR 10–12 min) with oxygen insufflation (*p* = 0.90). Median peak pCO_2_ was 77 mm Hg (IQR 70–84 mm Hg) with mechanical ventilation versus 74 mm Hg (IQR 70–82 mm Hg) with oxygen insufflation (*p* = 0.49). No patient demonstrated spontaneous respiratory effort during the apnea test. Among the 17 patients with direct neurointensivist supervision of the apnea test with real-time review of scalars during the QA period, no artifactual waveforms that resembled spontaneous respiratory effort or autotriggering occurred. No artifactual waveforms or autotriggering were documented in the medical record for the remainder of the apnea tests performed on mechanical ventilation (without real-time review by a neurointensivist). Oxygen desaturation to SpO2 < 90% did not occur during any apnea tests performed on mechanical ventilation but did occur during 4 of 32 (12.5%) apnea tests performed with oxygen insufflation (*p* = 0.01). The *P*/*F* ratios of these four patients before the apnea test were 64 mm Hg, 164 mm Hg, 268 mm Hg, and 400 mm Hg. Hypotension to MAP < 65 mm Hg despite titration of vasopressor was observed during 3 of 58 (5%) apnea tests performed on the ventilator and 4 of 32 (12.5%) tests with oxygen insufflation (*p* = 0.24). All patients with oxygen desaturation and hypotension (other than the patients previously described with unsuccessful apnea tests) achieved institutional pCO_2_ criteria for adequacy of respiratory stimulus, and successfully completed the apnea test. No patient suffered pneumothorax, arrhythmia, or cardiac arrest during apnea testing. Overall, instability of any kind during the apnea test occurred in 3 of 58 (5.2%) patients tested on mechanical ventilation and 7 of 32 (22%) patients tested with oxygen insufflation (*p* = 0.03). In multivariate analysis with patient instability of any cause as the binary response variable, only the *P*/*F* ratio before the apnea test (OR 0.98, 95% CI 0.97–0.99) and use of oxygen insufflation as the apnea test technique (OR 37.74, 95% CI 2.74–520.14) achieved statistical significance.

## Discussion

In our study, apnea testing performed on conventional mechanical ventilation was feasible and associated with less cardiopulmonary instability than the oxygen insufflation technique. This was despite preferential selection of the highest risk patients, such as those with ARDS (including all patients with COVID-19) and/or a requirement for ECMO, to undergo testing on mechanical ventilation by clinicians during the study period (Table 2), suggesting our study may underestimate the magnitude of benefit. Our finding is consistent with that of the 2019 Polish study, which also demonstrated lower drops in oxygenation with the mechanical ventilation technique [9], and with studies of CPAP valve-based techniques [7, 8]. No patient who underwent the apnea test on mechanical ventilation suffered oxygen desaturation, and only one apnea test on mechanical ventilation was unsuccessful, in the setting of severe hemodynamic instability. In addition to demonstrating the superior safety of this technique, our study is novel in being the only study of apnea testing performed on conventional mechanical ventilation as an integral part of the brain death evaluation process. In the 2019 Polish study, ventilator-based apnea testing was not used to determine brain death [9]. Sixty patients who had already been declared brain dead were selected on the basis of successful apnea test completion using the traditional technique. Patients who failed the traditional technique were excluded, potentially selecting study participants at a lower risk of cardiopulmonary instability. In our study, 24% of patients met criteria for ARDS and 84% required vasopressor support, reflecting a higher acuity referral population at increased risk for cardiopulmonary instability during the apnea test. Most patients with loss of brainstem function and vasomotor tone require vasopressor support and are at risk of further hemodynamic instability. Our study is also reassuring in demonstrating the absence of autotriggering events or artifactual waveforms resembling respiration with conventional mechanical ventilation. Although the ability to perform apnea tests on conventional ventilators is not new to many clinicians, and guidelines have endorsed the use of this technique, real-world data are essential to demonstrate the feasibility and relative merits of this technique.

**Fig. 1:**
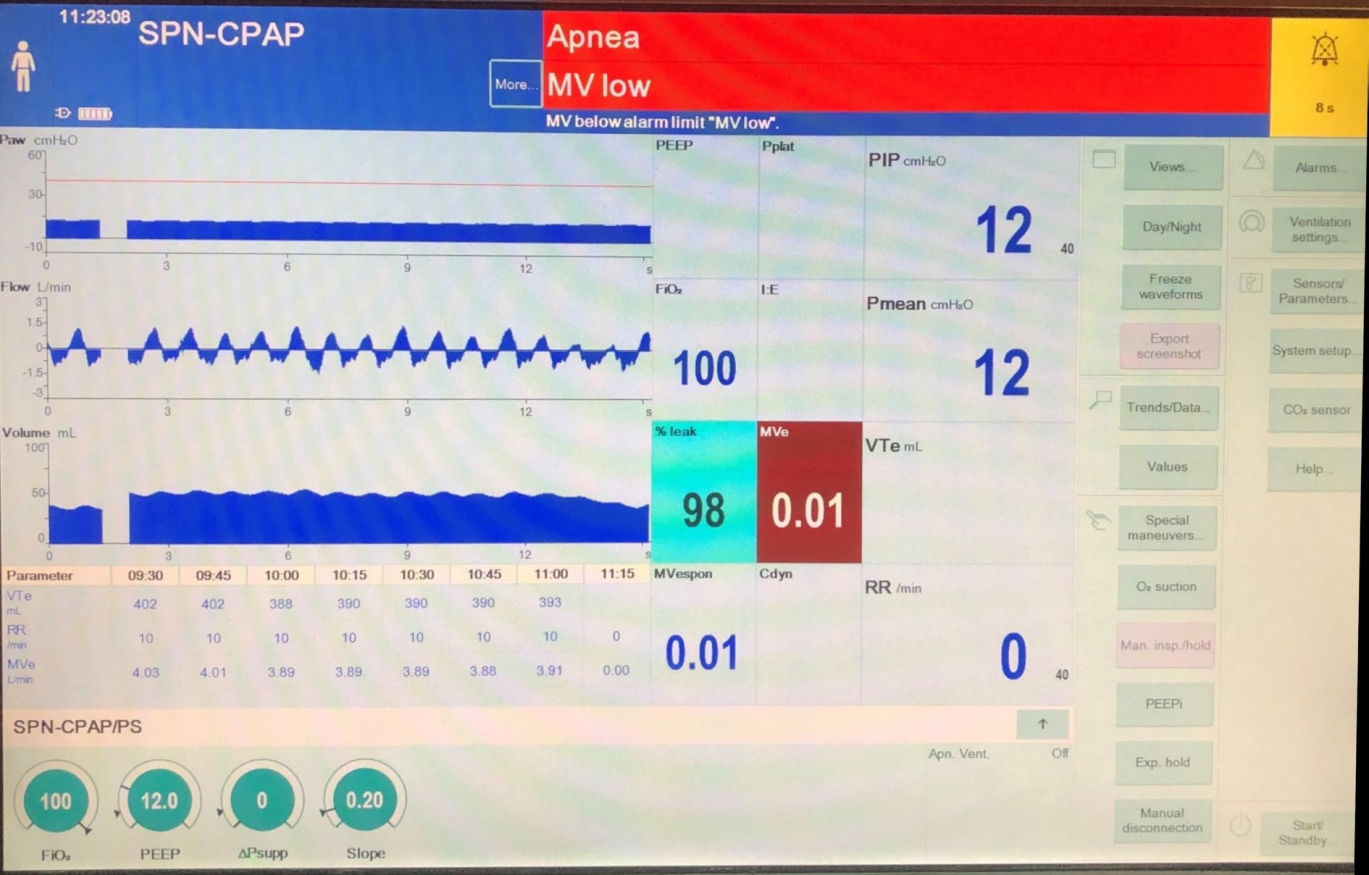
Scalar waveforms on a Dräger Evita Infinity V500 ventilator during apnea testing for evaluation of brain death. Cardiac oscillations are visible, without evidence of spontaneous respiratory effort. *CPAP* continuous positive airway pressure, *MV* mechanical ventilation, *SPN* spontaneous

Other studies have used improvised equipment, such as T-piece circuits and resuscitation bags connected to CPAP valves (Table 3) [7, 8, 12, 13]. These techniques require disconnection from the ventilator circuit and are therefore disadvantageous compared with the use of a conventional mechanical ventilator that is already supporting the patient. In addition, patients with ARDS may require higher PEEP to maintain oxygenation than feasible with many CPAP valves. These benefits were particularly highlighted during the COVID-19 pandemic, which posed specific challenges to the performance of apnea tests [14]. Disconnections of the ventilator circuit and oxygen insufflation increased the risk of viral transmission to health care providers, whereas high PEEP was frequently required to sustain oxygenation in the presence of severe ARDS. The use of conventional ventilators also eliminates the added risk, however small, of pneumothorax caused by oxygen insufflation through an oversized cannula, although pneumothorax may otherwise occur in any patient on positive pressure ventilation. Finally, ventilator scalars permit easy detection of subtle spontaneous respiratory effort, which may otherwise be missed through visual observation of the chest wall alone (Fig. 1). Our findings suggest that patients on ventilators that allow the apnea backup to be turned off should undergo apnea testing on the ventilator already in use, rather than with the traditional oxygen insufflation technique, given the confluence of evidence that now exists supporting superiority in maintaining cardiopulmonary stability with PEEP/CPAP. The need to minimize cardiopulmonary instability may be particularly relevant in the context of ethical concerns that some have raised about performing a procedure (the apnea test) that involves some risk without informed consent from surrogates [15]. We agree with the 2019 American Academy of Neurology position statement that suggests routine informed consent is not necessary, but that physicians have an obligation to minimize the risks of the apnea test, with standardized protocols [16]. It should be noted that several contemporary ventilators allow the use of this technique. Table 1, although not comprehensive, lists several commonly used ventilators and identifies models that allow disabling the apnea backup, along with the process for doing so.

**Table 3:** Published studies of apnea testing performed with CPAP/PEEP

First author	PMID	Region/country	Year	Sample size	Intervention/comparison	Outcome
Levesque	16,540,953	Quebec, Canada	2006	20	Prospective randomized crossover study of 3 techniques—oxygen catheter at 6 L/min, T piece 12 L/min, and CPAP with PEEP of 10 cm H20, and O2 of 12 L/min	No significant difference (*p* 0.96) between techniques in the increase in PaCO2 at the end of the apnea test. Fall in paO2 lower with CPAP than other groups (*p* < 0.01). In two patients, apnea testing could not be completed with an oxygen catheter and the T piece because of desaturation, although it could be completed with the CPAP
Giani	26,556,611	Monza, Italy	2015	169	Apnea test performed with ambu bag with PEEP valve with 8 L/min of oxygen. Non-ECMO patients were compared with ECMO patients	No interruption of apnea testing was noted and no complications
Kramer	28,176,180	Alberta, Canada	2017	77	Apnea testing was performed with an oxygen cannula at 5–8 L/min compared with CPAP valve set at 10 or the preexisting PEEP level	One test was aborted in each group, with no significant differences in PO2 reduction, rate of pCO2 rise, or pH decline
Park	31,083,250	Seoul, South Korea	2019	64	Apnea testing with oxygen cannula 15 L/min compared with ambu bag with PEEP valve	No significant changes were noted in PaCO2, PaO2, and pH. Subgroup analysis showed a dramatic reduction of PaO2 and SaO2 (*p* < 0.05) with oxygen cannula compared to ambu bag with PEEP in overweight patients and in patients with hypoxemic brain injury due to hanging
Solek-Pastuszka	30,209,714	Szczecin, Poland	2019	60	Apnea test with an oxygen cannula at 6 L/min was performed in all patients followed by apnea testing with PEEP at 10 cmH2O delivered via a conventional ventilator	Apnea test with oxygen cannula and with conventional mechanical ventilation were completed in all 60 patients without serious complications. Lower drop in paO2 with ventilator than oxygen cannula
Merchant		Michigan, USA	2024	92	Apnea testing completed on oxygen cannula versus mechanical ventilation	Apnea tests could not be completed successfully in 3/92 (3%)- 2 with oxygen insufflation (one case each with hypoxemia and hypotension) and 1 on mechanical ventilation (hypertension). Hypoxemia in 4/32 (12.5%) with oxygen insufflation and zero on mechanical ventilation (*p* = 0.01). Hypotension in 2/58 (3%) tests with mechanical ventilation and 4/32 (12.5%) with oxygen insufflation (*p* = 0.18). In multivariate analysis, the use of oxygen insufflation was an independent predictor of patient instability (odds ratio 37.74, 95% confidence interval 2.74–520.14)

Published studies of apnea testing performed with continuous positive airway pressure (CPAP) or positive end expiratory pressure (PEEP)

Our study has several limitations, primarily its retrospective nature and relatively small sample size, although it is the second largest study of PEEP/CPAP–based techniques to date (Table 3). The study was nonrandomized, and the ventilator technique was preferentially used in patients with poor oxygenation (Table 2), likely blunting the observed benefits of ventilator-based testing. Data on progressive changes in oxygenation and pCO_2_ through the course of the apnea test were unavailable.

In conclusion, apnea testing on conventional mechanical ventilation is feasible and offers several potential advantages over other techniques.
